# Electronic informed consent

**DOI:** 10.1186/1745-6215-14-S1-P91

**Published:** 2013-11-29

**Authors:** Natasha Stevens

**Affiliations:** 1 Pragmatic Clinical Trials Unit, London, UK

## 

Informed consent is a critical step in the recruitment of participants to any clinical trial and is essential to ensure participants are properly informed about the purpose of research, how it will be conducted, what is involved, the risks and benefits and rights to withdraw. The informed consent process is heavily regulated to ensure that the rights and wellbeing of trial participants is protected. In the world of electronic media and smart apps, is there a place in UK clinical trials for utilising electronic informed consent? I will report on the current views of research professionals and explore whether electronic informed consent forms (e-ICF) provide the same level of protection for participant's rights and wellbeing. I will address the regulatory aspects to be considered and the impact of e-ICF on data protection and confidentiality, as well as centralised trial monitoring for the clinical trials unit. I will focus on the types of trials where electronic informed consent may be appropriate and when it should be avoided. These are the areas that need to be addressed before electronic informed consent can be widely implemented by a clinical trials unit. In future work I will develop a patient focus group to explore e-ICF with patients and the likely impact on recruitment and retention rates and the role of the researcher in the e-ICF process.

